# Associations of Estimated Pulse Wave Velocity with Body Mass Index and Waist Circumference among General Korean Adults

**DOI:** 10.3390/metabo13101082

**Published:** 2023-10-15

**Authors:** Hack-Lyoung Kim, Hyun Sung Joh, Woo-Hyun Lim, Jae-Bin Seo, Sang-Hyun Kim, Joo-Hee Zo, Myung-A Kim

**Affiliations:** Division of Cardiology, Department of Internal Medicine, Seoul Metropolitan Government-Seoul National University Boramae Medical Center, Seoul National University College of Medicine, Seoul 07061, Republic of Korea; wingx4@naver.com (H.S.J.); woosion@gmail.com (W.-H.L.); cetuximab@naver.com (J.-B.S.); shkimmd@snu.ac.kr (S.-H.K.); jooheezo@hanmail.net (J.-H.Z.); kma@snu.ac.kr (M.-A.K.)

**Keywords:** arterial stiffness, body mass index, pulse wave velocity, obesity, waist circumference

## Abstract

The correlation between body fat parameters and arterial stiffness is still under debate. This study aimed to examine the associations of body mass index (BMI) and waist circumference (WC) with estimated pulse wave velocity (ePWV). We utilized data from 14,228 subjects (mean age 53.4 ± 16.8 years; 56.9% were female) from the Korean National Health and Nutrition Examination Survey. The ePWV was calculated using a formula based on age and blood pressure. Simple linear correlation analyses revealed significant associations between both BMI and ePWV (*r* = 0.098; *p* < 0.001) and WC and ePWV (*r* = 0.291; *p* < 0.001), with a stronger correlation observed between WC and ePWV. Multiple linear regression analysis demonstrated that WC remained significantly associated with ePWV after adjusting for potential confounders (*β* = 0.020; *p* = 0.001). However, a statistically significant association was not found between BMI and ePWV (*β* = 0.011; *p* = 0.076). Multiple binary logistic regression analysis further indicated that both higher BMI and WC were independently associated with higher ePWV, but the association was more pronounced between WC and ePWV than between BMI and ePWV. These findings underscore a stronger correlation between visceral obesity (as indicated by WC) and arterial stiffness (as indicated by ePWV) compared to overall obesity (as indicated by BMI). This highlights the potential significance of abdominal obesity in assessing cardiovascular risk.

## 1. Introduction

The relationship between obesity, specifically heightened body mass index (BMI), and cardiovascular disease (CVD) and mortality is well-established. While the relationship is not strictly linear, numerous studies have demonstrated that an increase in BMI is associated with an elevated risks of cardiovascular and cerebrovascular diseases [1,2,3,4]. With the surge in global obesity rates, examining CVD, a grave consequence of obesity, has become paramount. Traditionally, BMI has been the principal metric for obesity, relating weight to height [2,3,4,5]. A meta-analysis comprising 97 prospective cohort studies, which collectively included 1.8 million participants, demonstrated that a 5 kg/m^2^ increase in BMI was associated with a 27% increase in the risk for coronary heart disease and an 18% increase in stroke in multivariable analyses [2]. However, it does not fully account for body fat distribution, notably the harmful visceral fat enveloping vital organs. This recognition is fostering a shift towards waist circumference (WC) as a primary measure, offering direct insight into abdominal obesity and, consequently, visceral fat [5,6,7]. WC is increasingly seen as potentially superior in evaluating obesity-induced cardiovascular risks [8,9,10,11]. For example, in a prospective cohort study of 44,636 women in the Nurses’ Health Study, among normal-weight women (body mass index, 18.5 to <25 kg/m^2^), WC ≥ 88 cm was significantly associated with a 3-fold increase in CVD mortality [10]. This adjustment in measurement techniques signifies a crucial step in the advancement of accurate obesity-related risk assessment, potentially leading to more effective intervention strategies and improved health outcomes.

Arterial stiffness, a precursor to cardiovascular anomalies, results predominantly from aging and enduring exposure to cardiovascular risk factors like high blood pressure (BP), hyperglycemia, and inflammation [12,13]. Understanding arterial stiffness is important as it is often an early marker of organ damage, imminent cardiovascular events, and elevated mortality [12,13,14,15,16]. In a meta-analysis of 17 longitudinal studies that evaluated aortic PWV and followed up with 15,877 subjects for 7.7 years, an increase in aortic PWV by 1 m/s corresponded to age-, sex-, and risk factor-adjusted risk increases of 14%, 15%, and 15% in total CV events, CV mortality, and all-cause mortality, respectively [14]. Several methods are available to measure arterial stiffness, with pulse wave velocity (PWV) being the most commonly used [17]. While specialized equipment is typically required to measure PWV, it can also be estimated using an individual’s age and BP data, eliminating the need for advanced tools. This estimation is known estimated PWV (ePWV) [18]. Numerous studies have demonstrated that ePWV correlates well with other arterial stiffness measures and effectively predicts a subject’s prognosis [18,19,20,21,22]. Kim et al. [22] conducted a study with 1030 community-dwelling Korean individuals aged between 40 and 69. They demonstrated that those in the fourth quartile of ePWV had a 7.5-fold increase in CVD compared to those in the first quartile of ePWV. Since ePWV allows for the estimation of PWV without complex equipment, its application is expected to expand further in clinical practice.

Understanding the factors that influence arterial stiffness is crucial for both risk stratification and the advancement of therapeutic interventions. Arterial stiffness, a pivotal component in cardiovascular health, has been extensively studied in the context of its association with obesity. However, the results of these investigations have yielded mixed findings, leading to a lack of consensus in the field [23,24,25,26,27,28,29,30]. Moreover, a significant gap in the existing research is the differentiation between abdominal obesity and overall obesity when exploring their potential influence on arterial stiffness [24,31,32,33]. Our study aims to bridge this gap. Drawing data from a substantial national cohort, we specifically sought to examine the relationships between ePWV and two prevalent metrics of obesity: BMI and WC. Based on our preliminary observations and the existing literature, we postulated that WC, which offers a measure of abdominal obesity, would correlate more strongly with ePWV than BMI, an indicator of overall adiposity.

## 2. Methods

### 2.1. Study Population

Our study retrospectively analyzed data from the 8th Korea National Health and Nutrition Examination Survey (KNHANES). Conducted between 2019 and 2021, this iteration of the KNHANES continued the national surveillance initiative that has been tracking the health and nutritional status of Koreans since 1998. KNHANES is a nationwide survey conducted by the Korea Centers for Disease Control and Prevention. The survey collects information on the health and nutritional status of the Korean population, aiming to provide data that can be used for health policy development, health promotion, and disease prevention. The KNHANES employs a rolling sampling design that involves multiple stages, stratification, and clustering to ensure a representative sample of the South Korean population. The survey includes questionnaires, health examinations, and nutritional assessments, offering a comprehensive view of the health and nutritional landscape in Korea. KNHANES data are open for free use by Korean researchers. Initially, a cohort of 22,559 participants was enrolled in the 8th KNHANES, out of which 18,511 were adults aged 20 years or older. Following the exclusion of 4223 participants due to the absence of values of WC and BP, a total of 14,288 subjects were finally included in the analysis. This research adhered to a protocol that underwent review by the Institutional Review Board (IRB) (approval number, 07-2023-24). The requirement for informed consent was waived by the IRB, taking into consideration the retrospective nature of the analysis on a large dataset of patient information.

### 2.2. Clinical Data

BMI is a standardized metric to evaluate an individual’s weight relative to height. It is calculated by taking the individual’s weight in kilograms (kg) and dividing it by the square of their height measured in meters (m^2^). Waist circumference (WC), which offers insights into abdominal fat distribution, was measured by trained personnel. Using a tape measure, the WC was gauged at the midpoint between the lowest rib and the top of the iliac crest (pelvic bone) when the individual took a relaxed breath. Measurers of WC involved in the KNHANES project have extensive experience and undergo consistent training. BP was measured by an experienced examiner employing an automatic sphygmomanometer. Three readings were taken, with the BP calculated as the average of the last two readings [34,35]. To ensure accuracy in BP measurements, the routine calibration of equipment and training of examiners were carried out. Device calibration is executed through the ‘five-step quality control process for BP devices’, which encompasses before-use, in-use, and after-use calibrations, performed by a manufacturer’s technician in accordance with the British Hypertension Society protocol [36]. The BP measurement device is routinely replaced every 5–6 years. Comprehensive health data were gathered from participants using standardized questionnaires, capturing vital information on hypertension, diabetes, dyslipidemia, smoking habits, and prior CVD occurrences. Participants were asked to abstain from eating for 12 h, after which venous blood was drawn from the antecubital fossa. These samples underwent meticulous analysis to gauge various health indicators, including white blood cell count, hemoglobin, creatinine, glucose, glycated hemoglobin, and uric acid. Lipid profiles were also determined, focusing on total cholesterol, low-density lipoprotein cholesterol, high-density lipoprotein cholesterol, and triglyceride levels. Beyond these physical measurements, the survey further delved into the participants’ medicinal habits. Specifically, the use of medications for managing hypertension, diabetes, and dyslipidemia was documented, offering a holistic view of the participants’ health status and management strategies.

### 2.3. ePWV Calculation

The calculation of ePWV was performed according to existing literature data [18]. Initially, the mean BP (MBP) was calculated using the formula: MBP = diastolic BP + [0.4 × (systolic BP-diastolic BP)]. Subsequently, ePWV was computed by incorporating the patient’s age and MBP into the following equation: ePWV (m/s) = 9.587 − (0.402 × age) + (4.560 × 0.001 × age^2^) − (2.621 × 0.00001 × age^2^ × MBP) + 3.176 × 0.001 × age × MBP) − (1.832 × 0.01 × MBP) [18].

### 2.4. Statistical Analysis

Continuous variables were displayed as mean ± standard deviation, and categorical ones as numbers and percentages (n%). The relationships between ePWV and both BMI and WC were assessed via Pearson correlation analysis. The results of this analysis were then visually represented using scatter plots to easily discern patterns and trends. The relationships between ePWV and both BMI and WC were determined using multivariable linear regression and binary logistic regression analysis. For the linear regression, several clinical covariates were adjusted, including age, sex, systolic BP, glycated hemoglobin, low-density lipoprotein cholesterol, smoking habits, glomerular filtration rate, and uric acid levels. On the other hand, the binary logistic regression analysis made adjustments for age, sex, hypertension, diabetes mellitus, smoking habits, glomerular filtration rate, and uric acid levels. These adjustments ensured a comprehensive understanding of how BMI and WC independently relate to ePWV, accounting for a range of potential confounding factors. It is crucial to adjust for these variables to achieve a more accurate and nuanced insight into the association between obesity metrics and arterial stiffness. In the multivariate linear analysis, multicollinearity was assessed using the variance inflation factor (VIF), and the VIF values for all independent variables were below 4 [37]. In the multivariate binary logistic regression analysis, the dependent variable was determined based on the median value of ePWV. BMI and WC were categorized into tertiles and used as independent variables. To assess the goodness of fit for the logistic regression model, we utilized the Hosmer–Lemeshow Goodness of Fit test, with all resulting *p*-values being greater than 0.05 [38]. All analyses were two-tailed, with a *p*-value less than 0.05 indicating statistical significance. All statistical analyses were conducted using SPSS version 26.0 (IBM Co., Armonk, NY, USA).

## 3. Results

The characteristics of the study subjects (n = 14,228) are displayed in Table 1. The average age was 53.4 ± 16.8 years, with females constituting the majority of the subjects (8133, 56.9%). The mean BMI and WC were 24.0 ± 3.6 kg/m^2^ and 84.2 ± 10.4 cm, respectively. Systolic and diastolic BP were within normal ranges. The average ePWV was recorded as 8.87 ± 2.27 m/s. The prevalence rates of hypertension, diabetes mellitus, dyslipidemia, and cigarette smoking were 27.1%, 11.3%, 22.6%, and 15.7%, respectively. All major laboratory findings fell within normal limits. The proportions of subjects taking anti-hypertensive, anti-diabetic, and anti-dyslipidemic medications were 24.9%, 10.5%, and 17.2%, respectively.

**Figure 1:**
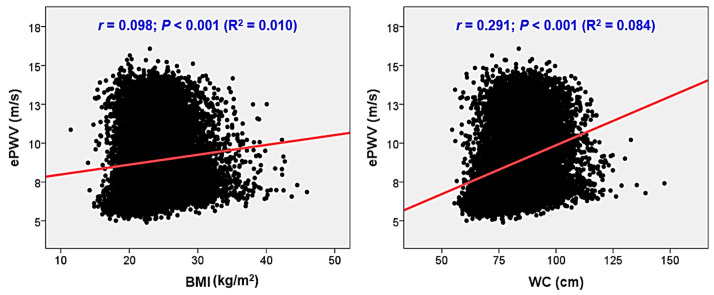
Scatter plots showing the correlations of ePWV with BMI and WC. ePWV, estimated pulse wave velocity; BMI, body mass index; WC, waist circumference.

Simple linear correlation analyses revealed significant associations between both BMI and ePWV (*r* = 0.098; *p* < 0.001), and WC and ePWV (*r* = 0.291; *p* < 0.001). However, the correlation strength was stronger between WC and ePWV compared to BMI and ePWV. This slight difference in associations between the two indicators can also be visually identified in the scatterplots (Figure 1). Multiple linear regression analysis illustrated that WC was significantly associated with ePWV even after controlling for potential confounders (*β* = 0.020; *p* = 0.001). However, this statistical significance was not observed in the association between BMI and ePWV (*β* = 0.011; *p* = 0.076) (Table 2). Multiple binary logistic regression analysis showed that both higher BMI (the lowest tertile vs. the middle tertile: odds ratio (OR): 1.38, 95% confidence interval (CI): 1.19–1.60, *p* < 0.001; the lowest tertile vs. the highest tertile: OR: 2.00, 95% CI: 1.70–2.34, *p* < 0.001) and WC (the lowest tertile vs. middle tertile: OR: 1.77, 95% CI: 1.52–2.05, *p* < 0.001; the lowest tertile vs. the highest tertile: OR: 2.76, 95% CI: 2.33–3.26, *p* < 0.001) were independently associated with higher ePWV (Table 3). However, the association was stronger between WC and ePWV than between BMI and ePWV.

## 4. Discussion

In this study, we examined the associations between BMI and WC with ePWV using data from the KNHEANS. We hypothesized that WC would exhibit a stronger correlation with arterial stiffness than BMI, and our results substantiate this hypothesis. The primary findings of our investigation are as follows: (1) Simple correlation analysis revealed that both BMI and WC were significantly correlated with ePWV; however, the correlation was notably stronger between WC and ePWV than between BMI and ePWV. (2) In the multiple linear regression analysis, the correlation between WC and ePWV remained significant, while the correlation between BMI and ePWV was not evident. (3) The multiple binary logistic regression analysis further confirmed that both BMI and WC were significantly associated with ePWV; yet, the association was more pronounced between WC and ePWV than between BMI and ePWV. Taken together, our findings suggest that the correlation was stronger between WC and ePWV than between BMI and ePWV. This outcome implies that visceral obesity, as indicated by WC, is more strongly associated with arterial stiffness than overall obesity, as indicated by BMI.

### 4.1. Association between Arterial Stiffness and Body Fat Parameters

The relationship between body fat parameters and arterial stiffness has produced mixed results in various studies. While some investigations indicate a positive correlation between body fat measures and arterial stiffness [28,30], others provide counterpoints with negative [23,29] or non-existent associations [26,27]. Numerous studies have proposed that BMI, an indicator of general obesity, and other body fat parameters suggesting visceral obesity, exhibit different relationships with arterial stiffness. A study involving 50 obese patients showed a connection between aortic PWV, as measured via magnetic resonance imaging, and BMI, but not with visceral fat area (VFA) [24]. Contrarily, a health check-up study of 11,061 subjects revealed a positive association between baPWV and waist/hip ratio, body shape index, and body roundness index, but not with BMI [33]. A similar trend was seen in another check-up study involving 2647 subjects where baPWV was associated only with body shape index and visceral adiposity index, but not with BMI [32]. A recent study involving 3758 subjects showed that baPWV was positively associated with VFA but not with BMI [31]. Our findings are consistent with these studies, demonstrating a stronger correlation between WC and ePWV, indicative of abdominal obesity, as opposed to BMI, indicative of overall obesity. A significant advantage of our study is the inclusion of the largest participant sample size, thus reinforcing the robustness of our results. Another distinguishing feature of our study is the use of ePWV as a measure of arterial stiffness. This calculated value, as opposed to a directly measured one, provides a more meaningful understanding of the link between arterial stiffness and abdominal obesity, further confirming the findings of prior studies.

### 4.2. BMI vs. WC

Although BMI is one of the most widely used anthropometric parameters, it has several shortcomings. BMI does not differentiate between muscle and fat mass, meaning muscular individuals may be classified as overweight or obese. BMI does not provide information on fat distribution within the body. Individuals with the same BMI may have different levels of visceral fat, which is more harmful. BMI does not consider age, sex, bone structure, and muscle mass, which can all affect body composition. Therefore, BMI may not be suitable for specific populations, such as athletes or older adults, due to muscle mass and bone density differences [39,40]. WC, on the other hand, provides a direct measure of abdominal fat, which is a significant risk factor for metabolic syndrome, type 2 diabetes, and CVD. High WC is associated with higher health risk, even if BMI is normal, allowing for more accurate risk stratification. Although WC has the disadvantage of measurement error and does not properly reflect fat distribution throughout the body, considering that it is more closely related to cardiovascular risk, it should be used more actively in classifying patients’ risk than BMI [8,9,10,11]. Since an increase in arterial stiffness is also closely related to CVD, our study results, which show that ePWV has a better correlation with WC than BMI, supports this suggestion.

### 4.3. The Usefulness of ePWV

Generally, PWV is determined by dividing the distance between two arterial points by the pulse wave transit time. In arteries with increased stiffness, PWV values correspondingly increase. Obtaining PWV via this direct measurement method necessitates specialized equipment and expertise [13]. ePWV, on the other hand, is derived from mathematical calculations and models instead of direct measurements, utilizing non-invasive data such as blood pressure, age, and heart rate. Different models may yield varying ePWV values. The method employed in this study is the most commonly used for ePWV, estimated using age and brachial BP. The ePWV calculation formula was obtained based on the numerous subjects’ carotid–femoral PWV (cfPWV) data [18]. cfPWV is regarded as the gold-standard non-invasive measure of arterial stiffness [41]. To obtain ePWV values, there is no need for specialized equipment or a trained examiner; patient age and BP measurements suffice. ePWV is invaluable in clinical settings where direct measurements are impractical and can be applied retrospectively to data where PWV was not measured. Its utility and validity have been substantiated in various studies, including those involving Asian populations [18,19,20,21,22], aiding in CVD risk stratification and managing conditions like hypertension. However, ePWV may not be as accurate as directly measured PWV due to the inherent limitations and assumptions of the estimation models. The clinical utility of ePWV should be continually validated across diverse populations and conditions.

### 4.4. Mechanisms

The exact pathophysiological underpinnings that explain the stronger correlation of WC with ePWV compared to BMI, as found in our study, are not entirely clear. Nevertheless, several potential mechanisms can be suggested. One key player in this context is visceral fat. Unlike subcutaneous fat, visceral fat actively secretes a plethora of inflammatory mediators. This leads to the amplification of insulin resistance, heightened oxidative stress, and endothelial cell dysfunction [6,42,43], all of which can induce structural alterations in the arterial wall, thus promoting arterial stiffness [44]. Another critical aspect to consider is the role of adipokines, which are bioactive peptides released by visceral adipose tissue with endocrine functions. For instance, leptin, an adipokine, is known to activate the sympathetic nervous system. This activation can lead to heightened BP and increased arterial stiffness. On the other hand, adiponectin, which has anti-inflammatory and anti-atherogenic properties, tends to be at reduced levels in individuals with visceral obesity [45]. Additionally, it is notable that visceral adipose tissue actively expresses and releases components of the renin–angiotensin system, like angiotensinogen and angiotensin II [46]. This system is well-acknowledged for its role in the onset of hypertension and vascular remodeling, both significant contributors to arterial stiffness. Furthermore, there are shared risk factors for both abdominal obesity and arterial stiffness, such as hypertension, diabetes mellitus, dyslipidemia, and sedentary lifestyles [17]. It is conceivable that these conditions, while augmenting arterial stiffness, might also have feedback mechanisms that indirectly contribute to abdominal obesity. In summary, while the intricate relationship between WC and ePWV is multifaceted, our understanding indicates that the influence of visceral adiposity on vascular health might be a pivotal factor. However, more in-depth research is required to unravel the full spectrum of mechanisms at play.

### 4.5. Clinical Implications

Our findings underscore the potential importance of abdominal obesity as a more substantial predictor for arterial stiffness, a condition associated with CVD. These results suggest that measuring WC could be an integral part of cardiovascular risk assessment. Moreover, these insights may assist healthcare providers in tailoring interventions designed to reduce visceral fat, such as diet modification, physical exercise, and potentially specific medications. Such strategies could help mitigate the risk of developing arterial stiffness and ensuing CVD. In addition, heightened awareness about the detrimental effects of abdominal obesity could inform public health strategies, prompting a concentrated effort towards preventing this type of obesity with the ultimate goal of reducing CVD prevalence. These findings may stimulate further research into the mechanistic links between abdominal obesity and arterial stiffness, possibly unveiling new therapeutic targets. The outcomes of this study might potentially shape future clinical guidelines and public health policies related to obesity and cardiovascular health. They emphasize the importance of monitoring WC along with BMI in the assessment and management of cardiovascular risk. Additionally, interventions aimed at improving abdominal obesity could be applied to ameliorate arterial stiffness. Evaluating the response to abdominal obesity treatment might involve tracking changes in arterial stiffness.

### 4.6. Study Limitations

Our study has several limitations. Firstly, the cross-sectional nature of our research design means we cannot definitively establish a cause-and-effect relationship between WC and ePWV. Secondly, we utilized ePWV, an estimated parameter of arterial stiffness, rather than a direct measure, which may affect the accuracy of our findings. Thirdly, we assessed obesity using only BMI and WC, overlooking other essential metrics like body fat percentage and visceral fat index. Furthermore, several potential confounding factors were not accounted for in our analysis. While we considered some variables, others such as dietary habits, physical activity levels, smoking status, alcohol consumption, and specific medical conditions were not adjusted for, which might have influenced the relationships we observed. Lastly, our study’s population consisted solely of Koreans. The specific genetic, dietary, and lifestyle factors inherent to this group mean our findings may not necessarily be generalizable to other ethnicities. As such, any attempt to apply our results to different populations should be approached with caution, considering potential differences in genetics, lifestyle, and environmental factors.

## 5. Conclusions

Our study reveals that ePWV demonstrates a stronger association with WC than with BMI. This supports the notion that arterial stiffness shares a closer association with visceral obesity than overall obesity. Additional studies are needed to further investigate the implications of these associations and explore potential interventions to mitigate the impact of abdominal obesity on arterial stiffness and, consequently, cardiovascular risk.

## Figures and Tables

**Table 1:** Characteristics of study subjects.

Characteristic	Value (n = 14,228)
Age, years	53.4 ± 16.8
Height, cm	163 ± 9
Weight, kg	64.3 ± 12.8
Body mass index, kg/m^2^	24.0 ± 3.6
Waist circumference, cm	84.2 ± 10.4
Systolic blood pressure, mmHg	119 ± 16
Diastolic blood pressure, mmHg	74.8 ± 9.8
ePWV, m/s	8.87 ± 2.27
Cardiovascular risk factors	
Hypertension	3867 (27.1)
Diabetes mellitus	1612 (11.3)
Dyslipidemia	3320 (22.6)
Cigarette smoking	2243 (15.7)
Previous myocardial infarction	181 (1.3)
Previous stroke	343 (2.3)
Laboratory findings	
White blood cell count, per µL	6130 ± 1720
Hemoglobin, g/dL	13.8 ± 1.5
Glomerular filtration rate, mL/min/1.73 m^2^	90.9 ± 19.2
Fasting glucose, mg/dL	102 ± 23
Glycated hemoglobin, %	5.83 ± 0.83
Uric acid, mg/dL	5.13 ± 1.38
Total cholesterol, mg/dL	190 ± 38
Low-density lipoprotein cholesterol, mg/dL	114 ± 36
High-density lipoprotein cholesterol, mg/dL	52.1 ± 12.7
Triglyceride, mg/dL	128 ± 97
Medications	
Anti-hypertensives	3554 (24.9)
Anti-diabetics	1057 (10.5)
Anti-dyslipidemics	2460 (17.2)

Numbers are expressed as mean ± standard deviation or n (%).

**Table 2:** Multiple linear regression analysis showing the associations of ePWV with BMI and WC.

Variable	*β*	t	*p*
BMI	0.011	1.778	0.076
WC	0.020	3.212	0.001

The following clinical covariates were adjusted during the analyses: age, sex, systolic blood pressure, glycated hemoglobin, low-density lipoprotein cholesterol, smoking, glomerular filtration rate, and uric acid. ePWV, estimated pulse wave velocity; BMI, body mass index; WC, waist circumference.

**Table 3:** Multiple binary logistic regression analysis showing the associations of ePWV with BMI and WC.

Variable	OR (95% CI)	*p*
*BMI*		
The lowest tertile (11.4~22.3 kg/m^2^)	1	-
The middle tertile (22.4~25.2 kg/m^2^)	1.38 (1.19–1.60)	<0.001
The highest tertile (25.3~45.9 kg/m^2^)	2.00 (1.70–2.34)	<0.001
*WC*		
The lowest tertile (54.5~79.4 cm)	1	-
The middle tertile (79.5~88.7 cm)	1.77 (1.52–2.05)	<0.001
The highest tertile (88.8~147.5 cm)	2.76 (2.33–3.26)	<0.001

The following clinical covariates were adjusted during the analyses: age, sex, hypertension, diabetes mellitus, smoking, glomerular filtration rate, and uric acid. ePWV, estimated pulse wave velocity; BMI, body mass index; WC, waist circumference.

## Data Availability

The data of the KNHANES is opened to the public, therefore, any researcher can be obtained after request from the following web-site: https://www.kdca.go.kr/yhs/yhshmpg/result/yhsresult/rawDtaList.do.

## References

[1] Powell-Wiley T.M., Poirier P., Burke L.E., Després J.P., Gordon-Larsen P., Lavie C.J., Lear S.A., Ndumele C.E., Neeland I.J., Sanders P. (2021). Obesity and Cardiovascular Disease: A Scientific Statement From the American Heart Association. Circulation.

[2] Lu Y., Hajifathalian K., Ezzati M., Woodward M., Rimm E.B., Danaei G. (2014). Metabolic mediators of the effects of body-mass index, overweight, and obesity on coronary heart disease and stroke: A pooled analysis of 97 prospective cohorts with 1.8 million participants. Lancet.

[3] Jee S.H., Sull J.W., Park J., Lee S.Y., Ohrr H., Guallar E., Samet J.M. (2006). Body-mass index and mortality in Korean men and women. N. Engl. J. Med..

[4] Whitlock G., Lewington S., Sherliker P., Clarke R., Emberson J., Halsey J., Qizilbash N., Collins R., Peto R. (2009). Body-mass index and cause-specific mortality in 900,000 adults: Collaborative analyses of 57 prospective studies. Lancet.

[5] Kim B.Y., Kang S.M., Kang J.H., Kang S.Y., Kim K.K., Kim K.B., Kim B., Kim S.J., Kim Y.H., Kim J.H. (2021). 2020 Korean Society for the Study of Obesity Guidelines for the Management of Obesity in Korea. J. Obes. Metab. Syndr..

[6] Després J.-P. (2021). Visceral Obesity with Excess Ectopic Fat: A Prevalent and High-Risk Condition Requiring Concerted Clinical and Public Health Actions. Cardiometab. Syndr. J..

[7] Ross R., Neeland I.J., Yamashita S., Shai I., Seidell J., Magni P., Santos R.D., Arsenault B., Cuevas A., Hu F.B. (2020). Waist circumference as a vital sign in clinical practice: A Consensus Statement from the IAS and ICCR Working Group on Visceral Obesity. Nat. Rev. Endocrinol..

[8] Bodenant M., Kuulasmaa K., Wagner A., Kee F., Palmieri L., Ferrario M.M., Montaye M., Amouyel P., Dallongeville J. (2011). Measures of abdominal adiposity and the risk of stroke: The MOnica Risk, Genetics, Archiving and Monograph (MORGAM) study. Stroke.

[9] Lee C.M., Huxley R.R., Wildman R.P., Woodward M. (2008). Indices of abdominal obesity are better discriminators of cardiovascular risk factors than BMI: A meta-analysis. J. Clin. Epidemiol..

[10] Zhang C., Rexrode K.M., van Dam R.M., Li T.Y., Hu F.B. (2008). Abdominal obesity and the risk of all-cause, cardiovascular, and cancer mortality: Sixteen years of follow-up in US women. Circulation.

[11] Teixeira J.E., Bragada J.A., Bragada J.P., Coelho J.P., Pinto I.G., Reis L.P., Fernandes P.O., Morais J.E., Magalhães P.M. (2022). Structural Equation Modelling for Predicting the Relative Contribution of Each Component in the Metabolic Syndrome Status Change. Int. J. Environ. Res. Public Health.

[12] Chirinos J.A., Segers P., Hughes T., Townsend R. (2019). Large-Artery Stiffness in Health and Disease: JACC State-of-the-Art Review. J. Am. Coll. Cardiol..

[13] Cavalcante J.L., Lima J.A., Redheuil A., Al-Mallah M.H. (2011). Aortic stiffness: Current understanding and future directions. J. Am. Coll. Cardiol..

[14] Vlachopoulos C., Aznaouridis K., Stefanadis C. (2010). Prediction of cardiovascular events and all-cause mortality with arterial stiffness: A systematic review and meta-analysis. J. Am. Coll. Cardiol..

[15] Ohkuma T., Ninomiya T., Tomiyama H., Kario K., Hoshide S., Kita Y., Inoguchi T., Maeda Y., Kohara K., Tabara Y. (2017). Brachial-Ankle Pulse Wave Velocity and the Risk Prediction of Cardiovascular Disease: An Individual Participant Data Meta-Analysis. Hypertension.

[16] Weber T. (2020). The Role of Arterial Stiffness and Central Hemodynamics in Heart Failure. Int. J. Heart Fail..

[17] Kim H.L., Kim S.H. (2019). Pulse Wave Velocity in Atherosclerosis. Front. Cardiovasc. Med..

[18] Greve S.V., Blicher M.K., Kruger R., Sehestedt T., Gram-Kampmann E., Rasmussen S., Vishram J.K., Boutouyrie P., Laurent S., Olsen M.H. (2016). Estimated carotid-femoral pulse wave velocity has similar predictive value as measured carotid-femoral pulse wave velocity. J. Hypertens..

[19] Ji C., Gao J., Huang Z., Chen S., Wang G., Wu S., Jonas J.B. (2020). Estimated pulse wave velocity and cardiovascular events in Chinese. Int. J. Cardiol. Hypertens..

[20] Vlachopoulos C., Terentes-Printzios D., Laurent S., Nilsson P.M., Protogerou A.D., Aznaouridis K., Xaplanteris P., Koutagiar I., Tomiyama H., Yamashina A. (2019). Association of Estimated Pulse Wave Velocity with Survival: A Secondary Analysis of SPRINT. JAMA Netw. Open.

[21] Wu L.D., Chu P., Kong C.H., Shi Y., Zhu M.H., Xia Y.Y., Li Z., Zhang J.X., Chen S.L. (2023). Estimated pulse wave velocity is associated with all-cause mortality and cardiovascular mortality among adults with diabetes. Front. Cardiovasc. Med..

[22] Kim B.S., Lee Y., Park J.K., Lim Y.H., Shin J.H. (2022). Association of the Estimated Pulse Wave Velocity with Cardio-Vascular Disease Outcomes among Men and Women Aged 40–69 Years in the Korean Population: An 18-Year Follow-Up Report on the Ansung-Ansan Cohort in the Korean Genome Environment Study. J. Pers. Med..

[23] Kim H.L., Lim W.H., Seo J.B., Kim S.H., Zo J.H., Kim M.A. (2022). Association between Body Mass Index and Arterial Stiffness. Cardiometab. Syndr. J..

[24] Rider O.J., Tayal U., Francis J.M., Ali M.K., Robinson M.R., Byrne J.P., Clarke K., Neubauer S. (2010). The effect of obesity and weight loss on aortic pulse wave velocity as assessed by magnetic resonance imaging. Obesity.

[25] Ounis-Skali N., Bentley-Lewis R., Mitchell G.F., Solomon S., Seely E.W. (2007). Central aortic pulsatile hemodynamics in obese premenopausal women. J. Am. Soc. Hypertens..

[26] Heleniak Z., Illersperger S., Brakemeier S., Dębska-Ślizień A., Budde K., Halleck F. (2020). Obesity, Fat Tissue Parameters, and Arterial Stiffness in Renal Transplant Recipients. Transplant. Proc..

[27] Rodrigues S.L., Baldo M.P., Lani L., Nogueira L., Mill J.G., Sa Cunha R. (2012). Body mass index is not independently associated with increased aortic stiffness in a Brazilian population. Am. J. Hypertens..

[28] Strasser B., Arvandi M., Pasha E.P., Haley A.P., Stanforth P., Tanaka H. (2015). Abdominal obesity is associated with arterial stiffness in middle-aged adults. Nutr. Metab. Cardiovasc. Dis..

[29] Tang B., Luo F., Zhao J., Ma J., Tan I., Butlin M., Avolio A., Zuo J. (2020). Relationship between body mass index and arterial stiffness in a health assessment Chinese population. Medicine.

[30] Wildman R.P., Mackey R.H., Bostom A., Thompson T., Sutton-Tyrrell K. (2003). Measures of obesity are associated with vascular stiffness in young and older adults. Hypertension.

[31] Kim H.L., Ahn D.W., Kim S.H., Lee D.S., Yoon S.H., Zo J.H., Kim M.A., Jeong J.B. (2021). Association between body fat parameters and arterial stiffness. Sci. Rep..

[32] Choi H.S., Cho Y.H., Lee S.Y., Park E.J., Kim Y.J., Lee J.G., Yi Y.H., Tak Y.J., Hwang H.R., Lee S.H. (2019). Association between new anthropometric parameters and arterial stiffness based on brachial-ankle pulse wave velocity. Diabetes Metab. Syndr. Obes..

[33] Zhang J., Fang L., Qiu L., Huang L., Zhu W., Yu Y. (2017). Comparison of the ability to identify arterial stiffness between two new anthropometric indices and classical obesity indices in Chinese adults. Atherosclerosis.

[34] Einstadter D., Bolen S.D., Misak J.E., Bar-Shain D.S., Cebul R.D. (2018). Association of Repeated Measurements with Blood Pressure Control in Primary Care. JAMA Intern. Med..

[35] Williams B., Mancia G., Spiering W., Agabiti Rosei E., Azizi M., Burnier M., Clement D.L., Coca A., de Simone G., Dominiczak A. (2018). 2018 ESC/ESH Guidelines for the management of arterial hypertension. Eur. Heart J..

[36] O’Brien E., Petrie J., Littler W., de Swiet M., Padfield P.L., Altman D.G., Bland M., Coats A., Atkins N. (1993). An outline of the revised British Hypertension Society protocol for the evaluation of blood pressure measuring devices. J. Hypertens..

[37] Marcoulides K.M., Raykov T. (2019). Evaluation of Variance Inflation Factors in Regression Models Using Latent Variable Modeling Methods. Educ. Psychol. Meas..

[38] Nattino G., Pennell M.L., Lemeshow S. (2020). Assessing the goodness of fit of logistic regression models in large samples: A modification of the Hosmer-Lemeshow test. Biometrics.

[39] Nevill A.M., Stewart A.D., Olds T., Holder R. (2006). Relationship between adiposity and body size reveals limitations of BMI. Am. J. Phys. Anthropol..

[40] Heymsfield S.B., Scherzer R., Pietrobelli A., Lewis C.E., Grunfeld C. (2009). Body mass index as a phenotypic expression of adiposity: Quantitative contribution of muscularity in a population-based sample. Int. J. Obes..

[41] Laurent S., Cockcroft J., Van Bortel L., Boutouyrie P., Giannattasio C., Hayoz D., Pannier B., Vlachopoulos C., Wilkinson I., Struijker-Boudier H. (2006). Expert consensus document on arterial stiffness: Methodological issues and clinical applications. Eur. Heart J..

[42] Caballero A.E. (2003). Endothelial dysfunction in obesity and insulin resistance: A road to diabetes and heart disease. Obes. Res..

[43] Kahn S.E., Hull R.L., Utzschneider K.M. (2006). Mechanisms linking obesity to insulin resistance and type 2 diabetes. Nature.

[44] Fantin F., Di Francesco V., Rossi A., Giuliano K., Marino F., Cazzadori M., Gozzoli M.P., Vivian M.E., Bosello O., Rajkumar C. (2010). Abdominal obesity and subclinical vascular damage in the elderly. J. Hypertens..

[45] Cheng K.H., Chu C.S., Lee K.T., Lin T.H., Hsieh C.C., Chiu C.C., Voon W.C., Sheu S.H., Lai W.T. (2008). Adipocytokines and proinflammatory mediators from abdominal and epicardial adipose tissue in patients with coronary artery disease. Int. J. Obes..

[46] Engeli S., Schling P., Gorzelniak K., Boschmann M., Janke J., Ailhaud G., Teboul M., Massiéra F., Sharma A.M. (2003). The adipose-tissue renin-angiotensin-aldosterone system: Role in the metabolic syndrome?. Int. J. Biochem. Cell Biol..

